# Persistent candidemia in very low birth weight neonates: risk factors and clinical significance

**DOI:** 10.1186/s12879-018-3487-9

**Published:** 2018-11-12

**Authors:** Jinjian Fu, Yanling Ding, Yongjiang Jiang, Shengfu Mo, Shaolin Xu, Peixu Qin

**Affiliations:** 1 Department of Laboratory, Liuzhou Maternity and Child Healthcare Hospital, 50th Yingshan Road, Chengzhong District, Liuzhou, 545001 China; 2 Department of Neonatology, Liuzhou Maternity and Child Health Care Hospital, Liuzhou, 545001 China

**Keywords:** Persistent candidemia, Very low birth weight, Neonates, Epidemiology

## Abstract

**Background:**

The prevalence and risk factors for persistent candidemia among very low birth weight infants are poorly understood. This study aimed to investigate the epidemiology of persistent candidemia over a 4-year period in a neonatal intensive care unit (NICU) in Liuzhou, China.

**Methods:**

We retrospectively extracted demographic data, risk factors, microbiological results and outcomes of very low birth weight infants with candidemia in our hospital between January 2012 and November 2015. Persistent candidemia was defined as a positive blood culture for > 5 days. Logistic regression was used to identify risk factors associated with persistent candidemia.

**Results:**

Of 48 neonates with candidemia, 28 had persistent candidemia. Both mechanical ventilation and intubation were significantly associated with increased rates of persistent candidemia (*P* = 0.044 and 0.004, respectively). The case fatality rate for the persistent candidemia group was 14.3%.

**Conclusion:**

The rate of persistent candidemia was high among very low birth weight neonates. Mechanical ventilation and intubation were the major factors associated with the development of persistent candidemia. This study highlights the importance of intensive prevention and effective treatment among neonates with persistent candidemia.

## Background

*Candida* species have emerged as important nosocomial infection pathogens associated with significant morbidity and mortality in very low birth weight (VLBW) neonates [1–4]. Candidemia is the third most common nosocomial bloodstream infection during late-onset neonatal sepsis [1]. It affects 10 to 20% of extremely low birth weight infants and 2 to 16% of very low birth weight neonates and is responsible for 25 to 30% of morbidity in neonatal intensive care units (NICUs) [2–7]. It was reported that the infants who survive candidemia frequently have long-term neurodevelopmental impairment, which occurred in 57% of these high-risk infants [7].

The contributing factors in high-risk groups include prematurity, VLBW, catheter and endotracheal tube use, prolonged NICU stay, broad-spectrum antibiotic use and total parenteral nutrition [8–10]. Lack of specific signs or symptoms in the development of candidemia among VLBW infants results were in a high risk of fatality [8]. Early diagnosis is crucial for infection control and initiating effective treatment.

Although VLBW is a well-known risk factor in the development of candidemia, it is uncertain whether this risk factor also contributes to persistent candidemia or mortality. Only a few studies have evaluated the risk factors and mortality for persistent candidemia in VLBW infants, and the results remain controversial. The potential risk factors and attributable mortality of persistent candidemia in VLBW infants in Western China are unknown. Therefore, we conducted this study to identify the incidence, risk factors, microbiological results and mortality associated with persistent candidemia in VLBW infants in China.

## Methods

### Data collection

A retrospective chart review of neonates admitted to the NICU of the Liuzhou Maternity and Child Healthcare Hospital from January 2012 to November 2015 was performed. Infants born at the hospital or transferred to the NICU at < 5 days old were included. At least one blood culture obtained by peripheral vein puncture that grew *Candida* species was defined as candidemia. Based on the study by Levy et al [11], if a single patient had a positive blood culture lasted for > 5 days starting from the first culture result indicated positive for *Candida*, the infection was considered persistent candidemia.

An electronic database was used to collect and record data. These included gestational age, birth weight, admission age, gender, delivery type, fetal membrane rupture duration, necrotizing enterocolitis, neurodevelopmental impairment, congenital diseases (such as congenital heart disease, glucose-6-phosphatedehydrogenase deficiency, and thalassemia), incidence of abdominal surgery, mechanical ventilation use, indwelling central venous catheter use, intubation > 6 days, rescue history, total parenteral nutrition status, hospitalization duration, 3rd generation cephalosporin use, carbapenem use, vancomycin use, antibiotic therapeutic duration, multiple antibiotic use (≥3 classes), *Candida* species, and mortality.

### Microbiologic methods

Blood cultures were incubated using the BacT/Alert 3D system (bioMerieux). *Candida* species were cultured in CHROM Agar medium (bioMerieux) and isolates were identified using API 20C AUX (bioMerieux).

### Statistical analysis

Statistical analysis was carried out using SPSS version 20.0 statistical software (SPSS Inc., Chicago, IL, USA). Potential risk factors associated with persistent candidemia were identified using logistic regression analysis. Variables with 2-tailed *P* < 0.05 were defined as statistically significant. Odds ratios (ORs) along with 95% confidence intervals (CIs) were used to assess the strength of any association.

## Results

### Incidence

During the 4-year period, a total of 5075 infants were admitted to the NICU, of which 484 were VLBW infants. A total of 48 cases among the VLBW infants were diagnosed with candidemia, resulting in a candidemia incidence of 9.5 per 1000 infants. Among the very low birth weight infants, the incidence of candidemia was 9.9%. For the 48 infants who suffered from candidemia, 28 experienced positive blood culture for > 5 days and were subsequently reported as having persistent candidemia. The persistent candidemia rate was 5.8% among VLBW infants.

Among the 48 *Candida* species, *Candida albicans* accounted for 39.6% of all cases (19/48), followed by *Candida glabrata* at 33.3% (16/48), and *Candida tropicalis* at 27.1% (13/48), no other *Candida* was found except *C*.*albicans*, *C. glabrata*, and *C. tropicalis*. *C*.*albicans* accounted for 25% (7/28) of cases of persistent candidemia. Non*-albicans* species were the leading causative pathogens of persistent candidemia, accounting for 75.0% of all cases, *C. glabrata* and *C. tropicalis* accounted for 42.9% (12/28) and 32.1% (9/28) of cases, respectively.

### Demographics

Among the 48 VLBW infants with candidemia, 17 were males and 31 were females. The median admission age was 3.2 days (range: 1.7–4.8 days), the median birth weight was 1154.5 g (range: 950.2 g − 1358.8 g), and the median gestational age was 29.6 weeks (range: 27.6–31.6 weeks) (Table 1).

**Table 1:** Clinical characteristics of neonates with and without persistent candidemia

Variable	Persistent candidemia		*P* value	Odds ratio (OR) (95% CI)
	Yes mean (95% CI) or *n* (%)	No mean (95% CI) or *n* (%)		
Demographics				
Gestational age (wks)	29.6 (27.3, 31.9)	29.7 (28.1, 31.2)	0.766	
Birth weight (g)	1097.8 (866.4,1309.2)	1229.0 (1051.1,1046.9)	0.029	
Male gender, *n* (%)	12 (42.9)	5 (25.0)	0.207	0.44 (0.13–1.57)
Admission age	1.3 (0.4, 2.2)	1.8 (2.4, 6.0)	0.611	
Risk factors				
Necrotizing enterocolitis	9 (32.1)	1 (5.0)	0.046	9.0 (1.04–78.17)
Neurodevelopmental impairment	6 (21.4)	6 (30.0)	0.501	0.64 (0.17–2.37)
Vaginal delivery	13 (46.4)	7 (35.0)	0.430	1.61 (0.49–5.25)
Fetal membrane rupture (h)	46.4 (25.1, 117.7)	18.8 (37.5, 75.1)	0.143	
Congentital diseases	15 (53.6)	10 (50.0)	0.867	1.15 (0.37–3.64)
Abdominal surgery	3 (10.7)	0 (0.0)	–	–
Mechanical ventilation	24 (85.7)	10 (50.0)	0.011	6.00 (1.52–23.70)
Central venous catheter	15 (59.6)	13 (65.0)	0.430	0.62 (0.19–2.03)
Intubation	20 (71.4)	3 (15.0)	0.000	14.17 (3.24–61.99)
Total parenteral nutrition	26 (92.9)	19 (95.0)	0.764	0.68 (0.06–8.11)
Hospitalization duration (d)	58.8 (33.8, 93.8)	49.3 (32.4, 66.2)	0.147	
3^rd^cephalosporins use	16 (59.3)	13 (65.0)	0.689	0.78 (0.24–2.59)
Carbapenems use	26 (92.9)	16 (80.0)	0.201	3.25 (0.53–19.82)
Vancomycin use	7 (25.0)	2 (10.0)	0.203	3.00 (0.55–16.31)
Multiple antibiotic use	18 (64.3)	13 (65.0)	0.959	0.97 (0.29–3.22)
Antibiotic therapeutic duration (d)	44.5 (24.3, 64.7)	31.1 (16.6, 55.6)	0.015	
Non-*C.albicans*	21 (75.0)	8 (40.0)	0.017	4.50 (1.31–15.52)
Prophylaxis antifungal therapy	25 (89.3)	16 (80.0)	0.375	2.08 (0.41–10.56)
Antifungal therapeutic duration (d)	11.3 (4.6, 18.0)	9.2 (4.6, 14.4)	0.243	
Outcome				
Death	4 (14.3)	1 (5.0)	0.320	3.17 (10.33–30.73)

### Clinical presentation

Most infants with candidemia received mechanical ventilation (70.8%, 34/48), central venous catheters (58.3%, 28/48), total parenteral nutrition (93.8%, 45/48), and had a rescue history (66.7%, 32/48) while 48% (23/48) of them received intubation for > 6 days. All of them received at least one class of antibiotics in the week before candidemia was diagnosed, and 64.6% received at least three classes of broad-spectrum antibiotics. The median antibiotic therapeutic duration was 38.9 days (range 20.7–58.0 days) while 85.4% of the neonates received prophylactic antifungal therapy with a median therapeutic duration of 10.4 days (range: 4.3–16.6 days). The median hospital length of stay was 54.9 days (range: 32.6–77.2 days).

### Risk factors

Compared to the non-persistent candidemia group, the persistent candidemia cases had a significantly lower birth weight [1229.0 vs. 1097.8 g; *P* < 0.001] and significantly longer antibiotic therapeutic duration [31.1 (16.6, 55.6) days vs. 44.5 (24.3, 64.7) days; *P* < 0.001]. Persistent candidemia infants also had higher incidence of necrotizing enterocolitis (32.1% vs. 5.0%; *P* < 0.001), mechanical ventilation (85.7% vs. 50.0%; *P* < 0.001), and intubation (71.4% vs. 15.0%; *P* < 0.001) than the non-persistent cases. The non-*albicans* species were found more frequently in the persistent candidemia group (75.0% vs. 40.0%; *P* < 0.001). A logistic regression analysis using backward model selection method on the 48 infants showed that candidemia cases on mechanical ventilation had a significantly increased risk of developing into persistent candidemia (OR = 5.72; 95% CI = 1.05–31.25), while intubation longer 6 days had an even higher risk (OR = 10.53; 95% CI = 2.11, 52.59) (Table 2).

**Table 2:** Multivariate analysis for persistent candidemia

Risk factor	Odds ratio	95% CI	*P* value
Intubation	10.53	2.11–52.59	0.004
Mechanical ventilation	5.72	1.05–31.25	0.044

### Outcome

The overall mortality among 484 VLBW infants was 10.4%. The mortality for the persistent candidemia group was 14.3%, and for those who experience none-persistent candidemia was 5.0%. This difference was not statistically significant (*P* = 0.320).

## Discussion

Candidemia remains a significant cause of morbidity and mortality in premature infants. The incidence of neonatal candidemia in our NICU (9.5 per 1000 infants) was similar to those found in many other studies, which reported incidences of candidemia near 10 per 1000 patient discharges [2, 4, 10].

It was remarkable that the majority (58.3%) of VLBW neonatal candidemia cases were persistent candidemia, which was similar to one study from Israel [11] that reported persistent candidemia in 52% of infants. Hammoud et al [12] identified that 60% of neonatal blood stream infections were persistent. The definition of persistent candidemia in the current study and the investigation was conducted by Levy et al [11] Although Robinson et al [13] reported an incidence of candidemia of 24.3% in a NICU located in the USA, their assessment of persistent candidemia incidence differed from Levy et al [11], which may explain the difference. The variation incidence highlights the importance of a consistent definition for persistent candidemia in neonates.

Many risk factors have been implicated in the pathogenesis of candidemia in infants, the most consistent one was prematurity, especially in very low birth weight groups [2, 5, 7, 8]. The results of the present study indicated that low birth weight was significantly associated with persistent candidemia, as revealed by univariate analysis, although no statistical significance was found by multivariate analysis. This finding was similar to a previous study [12], which also showed that the lower the birth weight, the greater the susceptibility to develop persistent candidemia (median birth weight, 970 and1130 g for the persistent candidemia and candidemia groups, respectively; *P* = 0.04).VLBW infants may have a higher risk of developing persistent candidemia because of their immature immune system, which may lead to an inability to eliminate pathogens from the bloodstream at the initiation of antifungal therapy.

Previous studies reported that *C.albicans* was the most frequent cause of candidemia in neonates, followed by *C. parapsilosis* [11]. Our study indicated that *C.albicans* was the predominant *Candida* species in VLBW infants, followed by Non- *albicans* such as *C. glabrata* and *C. tropicalis*. This pattern was consistent with previous reports conducted by Chinese, the American and Australian neonatal research groups [7, 9, 14–17], suggesting different epidemiology for neonatal candidemia around the world. A significantly higher frequency of non-*albicans* species (42.9 and 32.1% for *C.glabrata* and *C.tropicalis*, respectively) was found among VLBW infants with persistent candidemia compared to *C.albicans* (25.0%) in the current study. Published data addresses the significance of widespread implementation of prophylactic or empirical antifungal therapy in NICUs and PICUs [14–19], which may change *Candida* ecology. The identified risk factor for *C.glabrata* infection may be the result of broad-spectrum antibiotic use, particularly anti-aerobic and azole use as the empirical coverage for Gram-negative, Gram-positive and Fungal organisms in pediatric populations [2, 9, 16, 19]. One previous study identified *C.glabrata* as a risk factor for the development of persistent candidemia [20]. Additionally, non-*albicans* species themselves may also play a critical role in the pathogenesis of persistent candidemia due to their notorious capabilities of adherence to foreign surfaces, high virulence, and inherent or potential resistance to fluconazole [2, 3, 9, 19, 21–23].

Antibiotic exposure and, more importantly, the choice of an anti-aerobic spectrum of activity for routine prophylaxis or empirical therapy was the strongest risk factor for candidemia [14, 16, 24]. Our study was consistent with a previous report which showed that receipt of broad-spectrum antibiotics in the 7 days prior to candidemia diagnosis was a strong risk factor in VLBW infants [25]. A significantly longer duration of preceding antibiotic use was observed among infants with persistent candidemia when compared to infants with one episode of candidemia in the current study. This was consistent with the findings of a USA-based neonatal research group, which showed that prolonged antibiotic exposure was associated with persistent candidemia [26]. It is remarkable that the majority of infants in this study received empirical therapy (87.5%) in the preceding month. Moreover, over 85.4% of infants received multiple classes of antibiotics, either concomitantly or sequentially. Our data was consistent with the study that confirmed antibiotic prophylaxis was a common practice in many NICUs in China and around the world [9, 16, 17, 19, 27]. This finding highlights the need to reduce antibacterial exposure and employ effective medical practices for infection control.

Multiple explanations for the development of candidemia in neonatal groups have been suggested, and the most convincing explanation was the use of medical catheters and mechanical ventilation. Invasive operations provide a portal of entry and adhesion for *Candida* species and may be responsible for horizontal transmission [28]. It was suggested that due to the *Candida* species’ ability to adhere to foreign materials, a residual fungal deposit might exist even after prompt removal of infected catheters or medical appliances; thus, it may take some time to eliminate the *Candida* species given that the antifungal therapy is effective [29]. Medical appliances, such as intubation tubes, may be the cause of persistent candidemia [18]. In the current study, both mechanical ventilation and intubation were significantly associated with persistent candidemia, as revealed by univariate and multivariate regression analysis. Previous studies have also shown that medical catheter retention increased the risk of candidemia death [30] (OR, 95% CI = 2.50, 1.06–5.91), and early removal of catheters significantly reduced candidemia-associated mortality (OR, 95% CI = 20.5, 3.9–106.5), suggesting that the implementation of appropriate medical practices, such as early removal of intubation, might improve the prognosis of VLBW infants with persistent candidemia.

Candidemia-related mortality rates in neonates range between 43 and 54% [31, 32]. The candidemia mortality rate in our study was 10.4%, and all occurred in the persistent candidemia group, which had a mortality rate of 17.9%. Our report was consistent with the investigation conducted by Levy et al [11], which showed that the crude mortality rate in newborn infants was 17.8%. However, some studies showed that persistent candidemia infections have higher mortality rates compared to non-persistent candidemia. There are several explanations about this discrepancy, one being that we failed to identify an apparent source of the persistent candidemia in the majority of cases, as only five catheter tips tested positive for Candida species (three of which had persistent candidemia). Another explanation was that we defined persistent candidemia without considering the effectiveness of the antifungal therapy, which may result in underestimation of the clinical relevance of persistent candidemia.

The present analysis was limited by the retrospective design and the study’s location in a single center. Additionally, the small sample size may have compromised the statistical power of the study. A larger, multicenter prospective study is required to identify additional risk factors, ascertain the burden of persistent candidemia, and determine the antifungal susceptibility profiles to help pediatricians employ the proper intensive prevention and treatment practices.

## Conclusions

In conclusion, the present data demonstrate that the incidence of persistent candidemia was high in the VLBW infants at Liuzhou Maternity and Child Healthcare Hospital, and mechanical ventilation and intubation appeared to be the crucial factors for the development of persistent candidemia. This study highlights the importance of intensive prevention and effective treatment among neonates with persistent candidemia.

## Abbreviations

95%CI: 95% confidence interval
NICU: Neonatal intensive care unit
OR: Odds ratio
VLBW: Very low birth weight
